# Early-Life Exposure to Non-Nutritive Sweeteners and the Developmental Origins of Childhood Obesity: Global Evidence from Human and Rodent Studies

**DOI:** 10.3390/nu10020194

**Published:** 2018-02-10

**Authors:** Alyssa J. Archibald, Vernon W. Dolinsky, Meghan B. Azad

**Affiliations:** 1 Max Rady College of Medicine, University of Manitoba, Winnipeg, MB R3E 3P5, Canada; umarchib@myumanitoba.ca; 2 Developmental Origins of Chronic Diseases in Children Network (DEVOTION), Children’s Hospital Research Institute of Manitoba, Winnipeg, MB R3E 3P4, Canada; VDolinsky@chrim.ca; 3 Department of Pharmacology and Therapeutics, University of Manitoba, Winnipeg, MB R3E 0T6, Canada; 4 Department of Pediatrics and Child Health, University of Manitoba, Winnipeg, MB R3A 1S1, Canada

**Keywords:** non-nutritive sweeteners, artificial sweeteners, low-calorie sweeteners, developmental origins of health and disease, obesity, infants, children, pregnancy, prenatal nutrition

## Abstract

Non-nutritive sweeteners (NNS) are increasingly consumed by children and pregnant women around the world, yet their long-term health impact is unclear. Here, we review an emerging body of evidence suggesting that early-life exposure to NNS may adversely affect body composition and cardio-metabolic health. Some observational studies suggest that children consuming NNS are at increased risk for obesity-related outcomes; however, others find no association or provide evidence of confounding. Fewer studies have examined prenatal NNS exposure, with mixed results from different analytical approaches. There is a paucity of RCTs evaluating NNS in children, yielding inconsistent results that can be difficult to interpret due to study design limitations (e.g., choice of comparator, multifaceted interventions). The majority of this research has been conducted in high-income countries. Some rodent studies demonstrate adverse metabolic effects from NNS, but most have used extreme doses that are not relevant to humans, and few have distinguished prenatal from postnatal exposure. Most studies focus on synthetic NNS in beverages, with few examining plant-derived NNS or NNS in foods. Overall, there is limited and inconsistent evidence regarding the impact of early-life NNS exposure on the developmental programming of obesity and cardio-metabolic health. Further research and mechanistic studies are needed to elucidate these effects and inform dietary recommendations for expectant mothers and children worldwide.

## 1. Introduction

Childhood obesity is a health issue of increasing concern worldwide. One in three North American children are overweight or obese, with comparable rates in other developed countries [1,2,3,4,5]. Similar trends are also emerging in low- and middle-income countries, particularly in parts of Asia, where the prevalence of overweight and obesity has increased consistently over the past three decades [4,5]. This is alarming because obesity in childhood is a risk factor for many chronic diseases later in life [6]. An increasing number of children are being diagnosed with obesity-related conditions even before reaching adulthood, including type 2 diabetes, hypertension, chronic kidney disease, and sleep apnea [7,8]. Obesity and its complications appear to be rooted in early life—perhaps even in utero, according to The Developmental Origins of Health and Disease (DOHaD) hypothesis [9,10,11,12], which postulates that prenatal and early postnatal exposures can “program” lifelong metabolism, weight gain, and other endocrine pathways [9].

Sugar intake is a nutritional exposure that is strongly associated with obesity among children and adults alike [8,13,14,15]; hence, sugar substitutes or “non-nutritive sweeteners” (NNS) are promoted as a healthy alternative [16,17]. Also known as ‘sugar replacements’, ‘zero-calorie sweeteners’, or ‘artificial sweeteners’, commonly used NNS include synthetic compounds (e.g., aspartame, sucralose, acesulfame K, saccharin) and sugar alcohols (e.g., xylitol), as well as plant-derived compounds (e.g., steviol glycosides).

Contrary to their intended benefits, NNS have been associated with potentially adverse effects on cardiometabolic health in adults [18,19]; however, few studies have examined NNS exposure during pregnancy and early childhood. Furthermore, few studies have been conducted outside of the United States, Canada, or the UK [19]. The global impact of NNS exposure during these critical developmental periods is therefore unclear, and remains a point of controversy in the literature and among health authorities [20,21,22]. While the American Dietetic Association states that NNS consumption is safe in children and pregnant women within acceptable intake limits [17], the US Institute of Medicine cites a paucity of evidence of NNS safety and suggests avoiding NNS use in childhood [23]. In this review, we provide a comprehensive overview of the current body of literature regarding prenatal and childhood exposure to NNS worldwide, and their potential effects on weight gain, body composition, and obesity in human and animal studies. We also discuss current challenges and future research priorities in this field.

## 2. NNS Use in Children and Pregnant Women

NNS consumption among children has increased in recent years. In the 2009–2012 US National Health and Nutrition Examination Survey (NHANES), 25.1% of American children reported consuming foods or beverages containing NNS—up from 8.7% in 1999 [24]. In addition, other studies have shown that only one in four parents could correctly identify foods and beverages that were sweetened with NNS, indicating that many adults and children are consuming NNS unintentionally [25]. The increasing consumption of NNS among children is likely a consequence of marketing campaigns that promote NNS as a healthy alternative to sugar in an effort to curtail the childhood obesity epidemic [26,27], and this appears to be a global phenomenon. In a 2011–2013 multi-national survey of 9–11-year-old children, the International Study of Childhood Obesity, Lifestyle and Environment (ISCOLE) reported that over 50% of children surveyed consumed diet soft drinks, and 6–7% consumed them daily [28]. This survey included 12 countries spanning a range of economic and human development (Australia, Brazil, Canada, China, Colombia, Finland, India, Kenya, Portugal, South Africa, United Kingdom, United States), although consumption rates were not reported for individual countries. Notably, NNS exposure may also occur in utero, since NNS are commonly used by pregnant women. In the Canadian CHILD cohort, 29.5% of expectant mothers reported NNS use during pregnancy [29], while 45.4% of pregnant woman in the Danish National Birth Cohort reported NNS consumption [30]. Data are lacking for NNS consumption during pregnancy in other countries.

## 3. NNS Exposure in Childhood and Obesity-Related Outcomes

There are relatively few studies examining the cardio-metabolic effects of NNS use in children [22] compared to adults [19]. Table 1 summarizes 19 human studies reporting on NNS use and obesity-related outcomes in children, classified by study design. The majority were conducted in high-income countries (one in the Netherlands, two in the UK, and 14 in the US), except for one South African study [31] and one multi-national survey [28].

### 3.1. Randomized Controlled Trials (RCTs)

To date, only five RCTs have examined the effects of NNS use in childhood and subsequent weight-related outcomes. In the largest of these trials (*n* = 641 primarily normal weight Dutch children), those who received one daily artificially sweetened beverage (ASB) for 18 months exhibited reduced weight gain and fat accumulation compared to those given one daily sugar sweetened beverage (SSB) [34]. While these findings demonstrate a potential benefit of replacing SSB with ASB, they do not address the impact of adding ASB compared to water or other unsweetened beverages. The America on the Move Family Study (*n* = 218 American families with overweight children) examined the effects of replacing dietary sugars with NNS, combined with increased physical activity for 6 months, and showed that children enrolled in the treatment group were more likely to maintain or reduce their BMI compared to controls [32]; however, with this trial design, the effects of NNS could not be separated from the established beneficial effects of physical activity. Beverage replacement was also evaluated as a weight control strategy by Ebbeling et al. [35] (*n* = 224 American overweight adolescents), who reported a lower BMI and weight gain trajectory over 1 year (but not 2 years) with their intervention; however, SSB were replaced with a combination of ASB or unsweetened beverages, again making it difficult to isolate the specific effects of NNS.

In contrast to the RCTs described above, Taljaard et al. showed that South African children (*n* = 414) who consumed ASB with or without micronutrient fortification had higher BMI z-scores than their counterparts who consumed SSB with or without fortification [31]. Finally, in a smaller RCT of American overweight girls (*n* = 32), Williams et al. offered diet soda as a ‘healthy’ snack option as part of a dietary intervention, and found no effect on body weight compared to girls on a similar diet who were offered regular soda [33]. Overall, there is a paucity of RCTs evaluating NNS in children, and it is difficult to draw conclusions from the existing evidence due to study design limitations and differences between study populations.

### 3.2. Prospective Cohort Studies

Additional evidence for the potential effects of NNS on child weight and BMI has emerged from observational studies. This topic has been examined in eight prospective cohorts where changes in BMI and other anthropometric measures were recorded for up to 12 years following NNS exposure assessments in children (Table 1). All were initiated before 1997, and all but one [41] were conducted in the USA. All studies controlled for baseline body composition as part of their longitudinal design and adjusted for diet quality or total energy intake, and some additionally controlled for socioeconomic status, physical activity or inactivity, and other potential confounders. Four studies [38,40,41,43] found a positive association between childhood consumption of NNS and subsequent anthropometric outcomes, while the other four [36,37,39,42] found no association.

In the US Growing Up Today Study (*n* = 16,771 American children age 9–14 years), boys consuming diet soda at baseline experienced greater increases in BMI over two years of follow up; however, this association was not seen in girls [40]. In a smaller study of younger American children (*n* = 164, age 8–9 years), Blum et al. found a similar positive association among children of both sexes [38]. In the UK ALSPAC cohort (*n* = 1203 children age 5–7 years), Johnson et al. found that ASB intake in early childhood was associated with increased fat mass later in childhood; however, the association was largely explained by BMI at baseline, possibly reflecting an ineffective weight-control strategy used by overweight children in this study [41]. Finally, Hasnain et al. performed the longest follow-up study on this topic, assessing diet over 12 years in 103 American children from the Framingham Children’s Study, finding a positive association between ASB consumption and increasing adiposity (skinfold thickness) over time; however, no associations with weight or BMI were observed [43]. Notably in this study, ASB intake was very low, and was combined with unsweetened beverages for analysis, making it difficult to separate the potential independent effect of ASB. The remaining 4 cohort studies [36,37,39,42] found no clear association between ASB consumption and subsequent change in BMI or related outcomes in American children. Thus, overall there is inconsistent evidence for the impact of NNS exposure in childhood from prospective cohort studies.

### 3.3. Cross-Sectional Studies

Six cross-sectional studies have examined NNS or ASB consumption and concurrent anthropometric measures in children [28,44,45,46,48] (Table 1). Forshee et al. [44] assessed beverage consumption by American children (*n* = 3331 children age 6–19 years) and found a positive association between ASB intake and BMI, consistent with a smaller study (*n* = 385 American children age 11–13 years) by Giammattei et al. [45]. Similarly, Laverty et al. examined the dietary patterns of 13,170 children in the UK and found that daily ASB consumption was associated with a higher body fat percentage and BMI at 11 years of age [47]. In the International Study of Childhood Obesity, Lifestyle and the Environment, involving 6162 children from 12 countries spanning a range of economic and human development, Katzmarzyk et al. found a sex-specific and dose-dependent positive association between diet soda consumption and BMI z-score, percent body fat, and odds of obesity in girls [28].

Most recently, using data from the 2009–2014 US National Health and Nutrition Examination Survey (NHANES), Sylvetsky et al. reported positive associations between NNS consumption and obesity. In adolescents (ages 12–19), odds of obesity were consistently higher in NNS consumers than non-consumers, while in children (ages 2–11) this association was specifically observed in boys and participants who identified as Hispanic [48]. Notably, this was the only study to examine NNS consumption from foods as well as beverages. Using earlier NHANES data from 1999, O’Connor et al. found no association between ASB intake and BMI among preschool children [46].

Taken together, these studies provide important information about NNS consumption patterns in children, and identify potentially concerning associations with body composition and obesity. However, it must be acknowledged that confounding by reverse causation is possible in these cross-sectional studies, where it is not possible to establish whether the observed associations reflect a causal effect of NNS on body composition, or unsuccessful attempts to use NNS as part of a weight loss or weight management strategy.

## 4. NNS in Pregnancy and Obesity-Related Outcomes in Offspring

Few studies have investigated the effects of prenatal NNS exposure on obesity-related outcomes in offspring (Table 2). Two recent studies in Canada [49] and Denmark [29] have reported a positive and apparently sex-specific association between daily ASB consumption during pregnancy and higher BMI-*z* scores in male offspring, while a third study in the US found no association [10]. 

Using data from the general population CHILD (Canadian Healthy Infant Longitudinal Development) birth cohort of 2686 Canadian mother-infant dyads, we were the first to identify an association between ASB consumption during pregnancy and infant BMI [49]. In this study, daily ASB consumption during pregnancy (the highest consumption category) was associated with a 0.20 standard deviation increase in infant BMI z-score at 1 year of age (adjusted 95% CI 0.02, 0.38) and a 2-fold higher risk of overweight (adjusted odds ratio 2.19, 95% CI 1.23, 3.88), compared to no ASB consumption. These associations were independent of maternal pre-pregnancy BMI, diet quality, gestational diabetes, and other potential confounding factors. Consistent with these findings, in a study of 918 mothers with gestational diabetes from the Danish National Birth Cohort, Zhu et al. found that daily ASB consumption during pregnancy was associated with a 0.59 standard deviation increase in child BMI *z*-score at 7 years of age (adjusted 95% CI: 0.23, 0.96) and a 1.9-fold increased risk of overweight/obesity (adjusted relative risk 1.93; 95% CI; 1.24, 3.01); however, in contrast to the CHILD cohort, no association was observed with BMI during infancy [30]. Interestingly, in both studies, the observed associations between maternal ASB consumption and offspring body composition were stronger in males than females; however, the mechanism behind these sex differences is not known.

In contrast to the research described above, Gillman et al. did not observe an association between ASB intake during pregnancy and anthropometric outcomes in offspring in mid-childhood (median age 7.7 years) among 1078 children born to mothers without gestational diabetes in the American Project Viva cohort [10]. This could be due to their analytical approach; they evaluated ASB consumption as a continuous variable, which assumes a linear association with offspring body composition, yet the preceding studies did not observe a linear association—rather, significant associations were only found when ASB intake exceeded one serving per day. It is also noteworthy that despite finding no (linear) association with maternal ASB intake, Gillman et al. performed a substitution analysis and showed that replacing SSB with water was beneficial, whereas replacement with ASB was not.

Two additional studies have reported on birthweight following prenatal NNS exposure, although neither addressed this relationship as a primary research question. Maslova et al. examined birth weight as a covariate when studying childhood asthma in the Danish National Birth Cohort (*n* = 60,466), and found no crude association between birthweight and maternal ASB consumption during pregnancy [49]. Nakai et al. [50] performed a randomized controlled trial (RCT) of xylitol gum during pregnancy and postnatally in Japan (*n* = 107) to examine the transmission of caries-causing bacteria from mother to child, and did not detect an effect on birthweight. Overall, there is very little evidence regarding the effect of NNS consumption in pregnancy on obesity-related outcomes in offspring, and studies with different designs and analytical approaches have reached different conclusions.

## 5. Evidence from Animal Studies

Prenatal and early-life exposure to NNS has also been examined in animal models, providing a complimentary view and in-depth analysis of the potential mechanisms mediating the effect of NNS on weight gain and body composition (Table 3). Some studies have examined prenatal NNS exposure while others have studied NNS exposure through lactation, since several NNS have been detected in breast milk, including saccharin, sucralose, and acesulfame-potassium [52].

### 5.1. Prenatal NNS Exposure

Collison et al. showed that exposing mice to aspartame in utero and throughout life (0.25 g/L in drinking water fed to dams and offspring) resulted in increased body weight, visceral fat deposition, and fasting glucose levels [56]. Mice in this experiment consumed an average of 55 mg aspartame per kg of body weight per day, approximating the maximum Acceptable Daily Intake for humans (50 mg/kg/day), which equates to 92 packets or 17 diet sodas for a person weighing 68 kg (150 lbs). Using the same dose and protocol, these authors also showed that aspartame-exposed male offspring experienced greater weight gain, elevated fasting blood glucose levels, and decreased insulin sensitivity, while females had significantly raised fasting glucose levels [55]. Notably, because NNS exposure was continued from the prenatal period into adulthood, it was not possible to distinguish the impact of prenatal versus postnatal exposure in these experiments.

In another study, using a higher dose of aspartame limited to the prenatal period (2.0 g/L in drinking water to dams only; 343 mg/kg/day), von Poser Toigo et al. demonstrated that the male rat offspring exposed to aspartame during gestation had increased weight gain compared to offspring exposed to saccharin (1.35 g/L; 232 mg/kg/day) or control conditions. These male offspring also exhibited hyperglycemia and hyperlipidemia (elevated total cholesterol and triglycerides) characteristic of cardiometabolic disease. Moreover, they were more likely to choose and consume sweet foods in adulthood, suggesting that prenatal aspartame exposure affected taste preferences in these offspring [53]. In contrast, Soffritti et al. exposed mice to aspartame from mid-gestation until adulthood (a range of doses up to 32,000 ppm in feed, resulting in exposures up to 3909 mg/kg/day), and found no effect on body weight [54]; however, adiposity and other metabolic parameters were not assessed as this study was primarily focused on cancer.

### 5.2. NNS Exposure through Lactation

Two studies have specifically addressed lactational NNS exposure in rodents by feeding NNS during the lactation period only (i.e., dams were not exposed during pregnancy and offspring were not exposed after weaning). Parlee et al. reported that male mice exposed to saccharin through lactation (30 g/L in drinking water fed to dams) had decreased fat mass, increased lean mass, and reduced glycemia, while female offspring had decreased body weight [57]. In contrast, another study where rat dams were fed sorbitol during lactation (achieving exposures from 0.15 to 150 mg/kg/day) found that offspring had increased weight gain at lower doses and liver toxicity at higher doses compared to controls [58].

Together, these experimental studies provide inconsistent evidence for the impact of prenatal (Section 5.1) and early life (Section 5.2) NNS exposure on offspring metabolism and weight gain. Moreover, only high-dose exposures have been studied (often exceeding acceptable daily intake levels for humans), and the impact of prenatal versus lactational versus post-weaning exposure is unclear.

## 6. Possible Mechanisms

**Figure 1:**
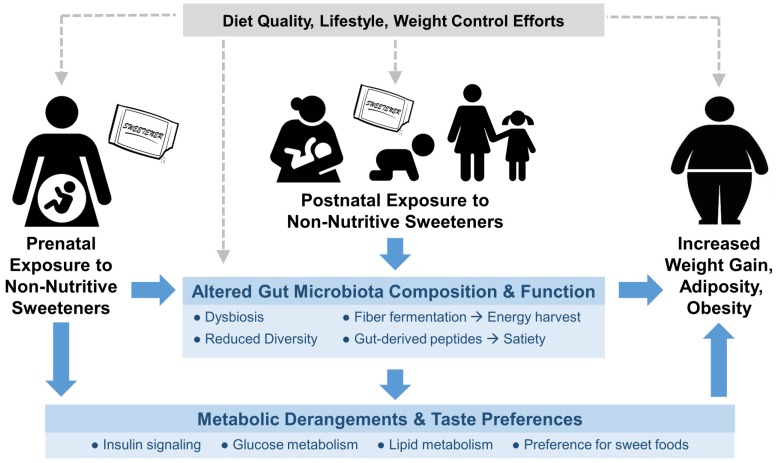
Conceptual framework for the impact of early-life exposure to non-nutritive sweeteners (NNS) on obesity-related outcomes later in life. Potential causal mechanisms are shown in blue; potential confounding factors are shown in grey. NNS exposure occurring in utero, through lactation, or via direct feeding may affect the developmental programing of metabolism, taste preferences, and gut microbiota, ultimately influencing weight gain, adiposity and obesity.

There are several potential mechanisms by which early-life NNS exposure may impact weight gain and body composition later in life (Figure 1), including the developmental programming of metabolism and taste preferences, metabolic hormone secretion, and disruption of gut microbiota [60]. These may result from direct exposure during infancy or childhood, or indirect exposure during gestation or lactation, since NNS are transferred in amniotic fluid and breast milk [52,61,62].

DOHaD research suggests that suboptimal maternal nutrition can predispose offspring to metabolic and cardiovascular disease, and may affect the development of adipose tissue in the fetus [63,64]. Routine NNS consumption has been associated with obesity-related impairments in glucose tolerance and energy homeostasis [19,65,66], which, when experienced during pregnancy, are consistently associated with increased odds of obesity among offspring [64,65,67]. Thus, regular NNS intake during pregnancy may program an adverse metabolic profile in the developing fetus, leading to increased weight gain and adiposity after birth.

Prenatal or early postnatal NNS exposure may also influence how the developing brain perceives sweet taste. While they have no nutritional impact, NNS trigger sweet taste receptors and routine exposure may alter thresholds for sweet taste perception, imparting a stronger preference for sweet foods later in life [20,60,68,69,70]. Regular consumption of NNS may alter reward-related regions in the brain, limiting the ability of the brain to predict the consequences of sweet sensation and blunting responses to caloric sweeteners [60,71,72]. NNS have also been shown to elicit metabolic responses independent of caloric load [73], including the secretion of incretin hormones that influence glucose sensing and glycemic control [60]. However, evidence remains inconsistent regarding the effect of NNS on gastric motility, gut hormones, or appetite responses in humans [74].

In addition, NNS may adversely impact the gut microbiota of mothers and their offspring. Most NNS are not directly digested by the consumer and therefore encounter the gut microbiota, which play an important metabolic and physiologic role in health and disease [75]. Intestinal microbes influence host weight gain by contributing to energy harvest from non-digestible foods and modulating glucose homeostasis and the release of gut-derived peptides that influence satiety and appetite regulation [76]. Although not yet confirmed in human studies, NNS have been shown to alter the gut microbiota in rodents, leading to impairments in glucose tolerance and the development of metabolic changes and obesity [77,78].

## 7. Limitations of Existing Studies and Knowledge Gaps Requiring Further Research

It is difficult to draw firm conclusions regarding the global impact of NNS during pregnancy and childhood due to the lack of data on consumption trends, inconsistencies between observational studies, paucity of evidence from low and middle-income countries, and lack of well-designed RCTs examining prenatal and early-life exposure to NNS. Limited studies in rodents provide complementary evidence on this topic, but these have rarely examined prenatal exposure separately from postnatal exposure, and most have used extreme doses that may not be relevant to humans.

While observational studies provide important evidence to inform new hypotheses and stimulate further research, results must be interpreted with caution due to the potential for confounding and the undefined temporality of exposure in cross sectional studies. Women may consume NNS in pregnancy or provide NNS to their children in an attempt to be healthy and avoid excessive weight gain, to compensate for a poor diet with excessive ‘junk foods’, or as a weight loss strategy if they are already overweight. Most studies adjust for these and other potential confounders, and many apply additional strategies to address reverse causality (e.g., excluding mothers or children who are obese at baseline), but residual confounding remains possible. Confounding is minimized in RCTs, but so far these have largely focused on ASBs without comparing them to unsweetened beverages, and have often involved multifaceted interventions, making it impossible to isolate the specific effects of NNS.

Existing studies are also limited by their NNS exposure assessments. Observational studies rarely distinguish between different types or sources of NNS, with most examining unspecified ASBs, ignoring the type of NNS and failing to capture NNS in foods. Notably, no human or animal studies have evaluated early-life exposure to the increasingly popular plant-derived NNS, such as stevia, which are commonly perceived to be “healthier” because they are extracted from natural sources. Moreover, most studies use self-report (or parent report) to assess NNS intake, which relies on participants to accurately identify NNS-containing products and accurately report their intake of these products. This is problematic because many consumers may be unaware of their NNS intake, leading to unreliable exposure estimates. Biomarkers can be used to quantitatively assess NNS intake [79]; however, very few studies have used these methods.

It is also important to note that most cohort studies have been conducted in high-income countries beginning in the 1990s, when NNS sources were relatively limited and consumption was much lower than it is today. To our knowledge, only one cross-sectional study [28] and two RCTs [31,34] have examined the relationship between NNS and childhood obesity outside of the US, Canada, or the UK. Therefore, it is unclear whether the reported findings are globally generalizable to other settings and/or contemporary populations. Finally, no existing studies have examined effects of early-life exposure beyond adolescence, or evaluated outcomes beyond body composition; thus, there is currently no evidence on the long-term impact of early-life NNS exposure on cardiometabolic health outcomes in adulthood.

Further research is needed to address the limitations of existing studies and critically evaluate the impact of early-life NNS exposure. A focus on accurately capturing NNS intake in beverages, foods, and other sources is required, and extended longitudinal follow up will be necessary to evaluate long-term effects. Choosing appropriate comparators in experimental studies and adjusting for relevant confounders in observational studies will help to establish the specific effects of NNS. Expanding research efforts beyond high-income countries, particularly in areas with increasing rates of obesity and/or NNS consumption, should be prioritized to address the global impact of NNS. In addition, studies are needed to confirm and explain the apparent sex-specificity of NNS effects, which have been inconsistently observed with childhood [28,40,48] and prenatal NNS exposures [29,30], and in rodent models [53,55,57]. Finally, mechanistic studies are required to establish causality, including human studies addressing biological mechanisms (e.g., incorporating microbiota analysis or metabolic profiling of mothers and offspring), as well as animal studies with physiologically relevant doses and precise exposure windows.

## 8. Conclusions

There is an emerging body of evidence from human and animal studies suggesting that early-life exposure to NNS may have adverse effects on cardio-metabolic health and development. However, current evidence remains inconclusive due to the paucity of RCTs, lack of evidence from low and middle-income countries, limitations of observational studies, and lack of mechanistic studies. Given the increasing popularity of NNS among all segments of the population, including pregnant women and children, further research is urgently needed to address this global knowledge gap. Considering the established detrimental effects of dietary sugars and the current uncertainty regarding NNS, limiting both is likely the most appropriate recommendation to pregnant women and children at this time, until higher quality evidence is available.

## Figures and Tables

**Table 1:** Summary of human studies evaluating non-nutritive sweetener (NNS) exposure during childhood and obesity-related outcomes.

Reference	Setting, Year, Study Name	Study Population	Age at Baseline	Duration of Follow Up	NNS Intervention or Exposure: Type, Measure, Method of Assessment	Confounders/Covariates Considered, and Comparators for RCTs	Outcomes Measured	Main Finding
**Randomized Controlled Trials**								
Rodearmel et al., 2007 [32]	USA, unspecified, AOM	218 families with overweight children	7–14 years	6 months	Personalized advice to increase physical activity and eliminate 100 kcal/day by replacing sugar in foods or beverages with NNS (sucralose); weekly self-report	Comparator: self-monitoring of physical activity and sweetened food and beverage consumption	BMI z-score, weight, % body fat, waist circumference	**Lower** odds of increasing BMI in intervention group
Williams et al., 2007 [33]	USA, unspecified	32 overweight girls	10–16 years	12 weeks	Calorie-restricted diet with diet soda offered, 24-h dietary intake by food diary (reviewed by RD)	Comparator: same diet with regular soda offered	Weight, BMI, blood pressure, total cholesterol, HDLC, triglycerides	**No difference** between groups
de Ruyter et al., 2012 [34]	Netherlands, 2009	641 primarily normal weight children	5–12 years	18 months	ASB, 1 can per day, daily consumption check by teacher	Comparator: SSB Covariates: age, sex, race; parent education, compliance, baseline values	BMI z-score, weight:height ratio, fat mass, skinfolds, waist circumference, % body fat	**Reduced** weight gain and fat accumulation with ASB vs. SSB
Ebbeling et al., 2012 [35]	USA, 2007	224 overweight and obese adolescents	14–16 years	2 years	Non-caloric beverages (ASB, unsweetened, water) to replace SSB, on demand, check-in meetings with participants	Comparator: no change in beverage consumption Covariates: age, sex, race; family income, parent education, physical activity, television viewing; baseline values	Change in BMI, weight	**Smaller** increase in BMI at 1 year (but not at 2 years) in intervention group
Taljaard et al., 2013 [31]	South Africa, 2010, BeForMi	414 children	6–11 years	10 months	Sucralose beverage with or without micronutrient fortification, 200 mL per day, daily consumption check by teacher	Comparator: SSB with or without micronutrient fortification Covariates: age, sex; baseline values	Weight, height, BMI z-scores	**Higher** weight for age z-score with ASB vs. SSB
**Prospective Cohort Studies**								
Ludwig et al., 2001 [36]	USA, 1995, Planet Health	548 children	12 ± 0.8 years	19 months	Diet soda, servings/day, Youth FFQ	Baseline BMI, total energy intake, physical activity, puberty, age, sex, race	BMI, obesity	**No association** with obesity
Newby et al., 2004 [37]	USA, 1995, ND WIC	1345 children	2–5 years	6–12 months	Diet soda, ounces/day, FFQ	Baseline BMI, total energy intake, change in height, socio-demographic status, age, sex	BMI, weight	**No association** of diet soda and change in weight or BMI
Blum et al., 2005 [38]	USA, 1992	164 children	8–9 years	2 years	Diet soda, 24-h diet recall	Baseline BMI, total energy intake, sex	BMI *z*-score, weight	**Positive** association of diet soda intake and BMI *z*-score change
Striegel-Moore et al., 2006 [39]	USA, 1987, NGHS	2371 girls	9–10 years	10 years	ASB, servings, 3-day food records	Baseline BMI, total energy intake, other beverages, age, race	BMI	**No association** of ASB and BMI change, though ASB associated with increased caloric intake
Berkey et al., 2007 [40]	USA, 1996, Growing Up Today	16,771 children	9–14 years	2 years	Diet soda, servings, FFQ	Baseline BMI, total energy intake, physical activity, screen time, puberty, age, sex, race	BMI (self-reported)	**Positive** association of diet soda intake and BMI gain in boys
Johnson et al., 2007 [41]	UK, 1991, ALSPAC	1203 children	5–7 years	2–4 years	Low-energy beverages, volume and/or servings/day, 3-day unweighted diet diaries	Baseline BMI, total energy intake, diet quality, height, television watching, socioeconomic status, parent BMI, sex	Fat mass index	**Positive** association of low-energy beverage intake with fat mass increase, mostly explained by baseline BMI
Kral et al., 2008 [42]	USA, unspecified	177 children	3–6 years	3 years	ASB, servings, 3-day food record	Baseline BMI, total energy intake, obesity risk status based on maternal BMI	BMI z-score, waist circumference	**No association** of change in ASB consumption and change in BMI
Hasnain et al., 2014 [43]	USA, 1987 FCS	103 children	3–9 years	12 years	ASB (combined with unsweetened beverages), ounces/day, 3-day records	Baseline BMI and body fat, % energy from fat, screen time, other beverage intakes, maternal education, maternal BMI, age	BMI, waist circumference, skinfolds, % body fat	**Positive** association of ASB intake and change in skinfold measurements; no association with weight or BMI change
**Cross-sectional Studies**								
Forshee et al., 2003 [44]	USA, 1994, USDA CSFII	3311 children and adolescents	6–19 years	-	Diet soda and diet fruit drinks, g/day, survey	Age, sex, race, family income	BMI	**Positive** association between diet soda (but not diet fruit drink) consumption and BMI
Giammattei et al., 2003 [45]	USA, 2000	385 children	11–13 years	-	Diet soda, number consumed/day, questionnaire	Race, physical activity, inactivity/screen time, family eating patterns	BMI *z*-score, % body fat	**Positive** correlation between diet soda consumption and BMI *z*-score/percent fat
O’Connor et al., 2006 [46]	USA, 1999, NHANES	1160 children	2–5 years	-	ASB, servings, 24-h dietary recall	Age, sex, ethnicity, family income, energy intake, physical activity	BMI percentile	**No association** of ASB intake and BMI
Laverty et al., 2015 [47] **	UK, 2008, MCS	13,170 children	7–11 years	-	ASB, servings/week, caregiver reporting	Age, sex, race, family income, maternal education, country, fruit consumption, breakfast consumption, physical activity, television watching	BMI, % body fat	**Higher** BMI and % body fat with daily ASB consumption
Katzmarzyk et al., 2016 [28]	12 countries * ISCOLE	6162 children	9–11 years	-	Diet soda, number per week, FFQ	Age, sex, study site, parent education, physical activity	BMI *z*-score, % body fat, obesity	**Positive** dose-dependent association of diet soda intake with BMI, % body fat and odds of obesity in girls
Sylvetsky et al. 2017 [48]	USA, 2009–2014, NHANES	9261 children and adolescents	2–19 years	-	Foods and beverages containing NNS, number of items, 24-h dietary recalls	Sex, race, family income, energy intake, physical activity	Obesity	**Higher** odds of obesity in boys consuming ASB and Hispanic participants consuming NNS. Higher odds of obesity with NNS consumption in adolescents.

Studies sorted by year of publication. Abbreviations: ALSPAC, Avon Longitudinal Study of Parents and Children; AOM, America on the Move; ASB, artificially-sweetened beverage; BeForMe, Beverage Fortified with Micronutrients; BMI, body mass index; DMFT, decayed, missing, and filled teeth; DNBC, Danish National Birth Cohort; FCS, Framingham Children’s Study; FFQ, food frequency questionnaire; GA, gestational age; MCS, Millennium Cohort Study; ND WIC, North Dakota Women and Children; NGHS, National Growth and Health; NHANES, National Health and Nutrition Examination Survey; NNS, non-nutritive sweetener; SES, socioeconomic status; SSB, sugar-sweetened beverage; USDA CSFII, United States Department of Agriculture Continuing Survey of Food Intake by Individuals.* Australia, Brazil, Canada, China, Colombia, Finland, India, Kenya, Portugal, South Africa, United Kingdom, United States. ** This longitudinal study evaluated ASBs in a cross-sectional manner because ASB data were only collected at the final assessment. **Bold** text indicates main direction of association between NNS exposure and obesity-related outcome.

**Table 2:** Summary of human studies evaluating non-nutritive sweetener (NNS) exposure during pregnancy and obesity-related outcomes in offspring.

Study, Year	Setting, Year of Study Enrollment/Baseline Intake, Study Name	*n*	Timing of Prenatal NNS Exposure	Duration of Follow Up	NNS Type, Measure, Method of Assessment	Confounders/Covariates Considered, and Comparators for RCTs	Outcomes in Offspring	Main Finding
**Randomized Controlled Trials**								
Nakai et al., 2008 [50]	Japan, unspecified	107 pregnant women	6th month of pregnancy to 9 months postpartum	13 months	Xylitol gum, 1 pellet at least 4x/day	Maternal age, oral examination (DMFT); child birthweight, sex. Comparator: no gum	Birth weight (examined as a covariate)	**No association** of infant birth weight and daily maternal xylitol gum
Maslova et al., 2013 [49]	Denmark, 1996, DNBC	60,466 pregnant women	Prenatal; 25th week pregnancy	7 years	ASB, servings, validated FFQ	Maternal BMI, total energy intake, parity, smoking, exercise, gestational weight gain, education and occupation, breastfeeding duration; child gestational age, sex	Birth weight (examined as a covariate)	**No association** of infant birth weight with maternal ASB intake
**Prospective Cohort Studies**								
Azad et al., 2016 [29]	Canada, 2009, CHILD	2686 pregnant women	Prenatal exposure	1 year	ASB, servings, validated FFQ	Maternal BMI, total energy intake, diet quality, age, education, smoking, diabetes; infant gestational age, sex, birth weight; breastfeeding duration, timing of solid food introduction	BMI z-score, overweight	**Higher** infant BMI and risk of overweight with daily maternal ASB consumption (males only)
Gillman et al., 2017 [51]	USA, 1999, Project Viva	1078 pregnant women without gestational diabetes	Prenatal exposure	6.6–10.9 years	ASB, servings, validated FFQ	Maternal BMI, age, race, education, smoking, parity; household income; child age, sex	Adiposity (BMI *z*-score, fat mass index, skinfolds), central adiposity (skinfold ratio, WC)	**No association** of child adiposity with maternal ASB intake
Zhu et al., 2017 [30]	Denmark, 1996, DNBC	918 pregnant women with gestational diabetes	Prenatal exposure	7 years	ASB, servings, validated FFQ	Maternal BMI, energy intake and diet quality, age, employment level, smoking, physical activity; infant sex, breastfeeding duration; child ASB/SSB consumption, physical activity	Large-for-gestational age (LGA), BMI *z*-score, overweight/obesity	**Higher** BMI and risk of LGA and overweight with daily maternal ASB intake (effect larger in boys)

Studies sorted by year of publication. Abbreviations: ASB, artificially-sweetened beverage; BMI, body mass index; CHILD, Canadian Healthy Infant Longitudinal Development; DMFT, decayed, missing, and filled teeth; DNBC, Danish National Birth Cohort; FFQ, food frequency questionnaire; GA, gestational age; NNS, non-nutritive sweetener; SES, socioeconomic status; SSB, sugar-sweetened beverage. **Bold** text indicates main direction of association between NNS exposure and obesity-related outcome.

**Table 3:** Summary of animal studies evaluating early-life non-nutritive sweetener (NNS) exposure and obesity-related outcomes.

Study, Year	Animal Model	NNS Type	NNS Dose, Route (Exposure) * to Dams	Timing of NNS Exposure	Outcomes Measured	NNS Effects in Offspring
**Prenatal Exposure only**						
von Poser Toigo et al., 2015 [53]	Wistar rats	Aspartame or Saccharin	Aspartame: 2 g/L in water ad libitum (343 mg/kg/day) ** or Saccharin: 1.35 g/L in water ad libitum (232 mg/kg/day) **	30 days pre-conception until birth	Body weight, metabolic profile, feeding behavior, anxiety	**Increased** weight gain, serum cholesterol and triglycerides, intake of sweet foods with aspartame exposure (stronger effects in males); increased weight gain with saccharin exposure (males only)
**Prenatal Exposure, Continued through Lactation and Post-weaning**						
Soffritti et al., 2010 [54]	Swiss mice	Aspartame	0–32,000 ppm in feed ad libitum (0–3903 mg/kg/day)	12th day gestation to natural death or 130 weeks of age	Neoplastic lesions, body weight (as a covariate)	**No difference** in body weight between consumption groups
Collison et al., 2012 [55]	C57BL/6J mice	Aspartame	0.25 g/L in water ad libitum (55 mg/kg/day)	3 weeks pre-conception to 17 weeks of age	Fasting blood glucose, insulin, lipid profile, body weight, % weight gain, visceral fat	**Increased** weight gain (males only), decreased insulin sensitivity; elevated fasting glucose levels (females only)
Collison et al., 2012 [56]	C57BL/6J mice	Aspartame	0.25 g/L in water ad libitum (55 mg/kg/day)	3 weeks pre-conception to 20 weeks of age	Glucose and insulin homeostasis, body weight, adiposity	**Increased** body weight and fasting blood glucose; decreased insulin sensitivity
**Exposure through Lactation only**						
Parlee et al. 2014 [57]	C57BL/6J mice	Saccharin	3% Saccharin (30 g/L) in water ad libitum (280 μM in serum of pups)	Birth to 21 days	Weight, body composition by NMR, adipocyte size and number, serum insulin concentration, glucose tolerance test	**Decreased** body weight (females only), increased lean and decreased fat mass (males only), increased small and decreased large adipocytes, improved glucose tolerance
Cardoso et al. 2016 [58]	Wistar rats	Sorbitol	Exact amount required to achieve target dose, diluted in 2 mL water (0.15 to 150 mg/kg/day)	Birth to 14 days	Weight gain, serum proteins, cholesterol, glucose, liver enzymes	**Increased** weight gain and total serum cholesterol with low dose; liver toxicity, lower serum glucose and triglycerides with high dose

Studies sorted by year of publication. Abbreviations: NMR, nuclear magnetic resonance; NNS, non-nutritive sweetener. * For comparison, the US FDA acceptable daily intakes for humans are 50 mg/kg/day for aspartame and 15 mg/kg/day for saccharin [59]. ** Exposure not reported by authors; estimated from reported dose, approximate consumption and body weight. **Bold** text indicates main direction of association between NNS exposure and obesity-related outcomes.
